# Relationship of triglyceride-glucose index with cardiometabolic multi-morbidity in China: evidence from a national survey

**DOI:** 10.1186/s13098-023-01205-8

**Published:** 2023-11-06

**Authors:** Zenglei Zhang, Lin Zhao, Yiting Lu, Xu Meng, Xianliang Zhou

**Affiliations:** Department of Cardiology, Fuwai Hospital, National Center for Cardiovascular Diseases, Chinese Academy of Medical Sciences and Peking Union Medical College, No.167, Beilishi Road, Xicheng District, Beijing, China

**Keywords:** Cardiometabolic multi-morbidity, TyG index, Cardiometabolic disease, Insulin resistance, Risk factors, CHARLS

## Abstract

**Background:**

Cardiometabolic multi-morbidity (CMM) is emerging as a global healthcare challenge and a pressing public health concern worldwide. Previous studies have principally focused on identifying risk factors for individual cardiometabolic diseases, but reliable predictors of CMM have not been identified. In the present study, we aimed to characterize the relationship of triglyceride-glucose (TyG) index with the incidence of CMM.

**Methods:**

We enrolled 7,970 participants from the China Health and Retirement Longitudinal Study (CHARLS) and placed them into groups according to quartile of TyG index. The endpoint of interest was CMM, defined as the presence of at least two of the following: stroke, heart disease, and diabetes mellitus. Cox regression models and multivariable-adjusted restricted cubic spline (RCS) curves were used to evaluate the relationship between TyG index and CMM.

**Results:**

In total, 638 (8.01%) incident cases of CMM were recorded among the participants who did not have CMM at baseline (2011) during a median follow-up of 84 months (interquartile range, 20‒87 months). The incidences of CMM for the participants in quartiles (Q) 1–4 of TyG index were 4.22%, 6.12%, 8.78%, and 12.60%, respectively. A fully adjusted Cox model showed that TyG index was closely associated with the incidence of CMM: the hazard ratio (HR) [95% confidence interval (CI)] for each 1.0-unit increment in TyG index for CMM was 1.54 (1.29–1.84); and the HRs (95% CIs) for Q3 and Q4 (Q1 as reference) of the TyG index for CMM were 1.41 (1.05–1.90) and 1.61 (1.18–2.20), respectively. The association of TyG index with the incidence of CMM was present in almost all the subgroups, and persisted in the sensitivity analyses and additional analyses. Multivariable-adjusted RCS analysis revealed a significant dose-response relationship of TyG index with the risk of CMM (overall *P* < 0.001; non-linear *P* = 0.129).

**Conclusions:**

We found that a high TyG index is associated with a higher risk of incident CMM. This finding may have significance for clinical practice and facilitate the creation of a personalized prevention strategy that involves monitoring the TyG index.

**Graphical Abstract:**

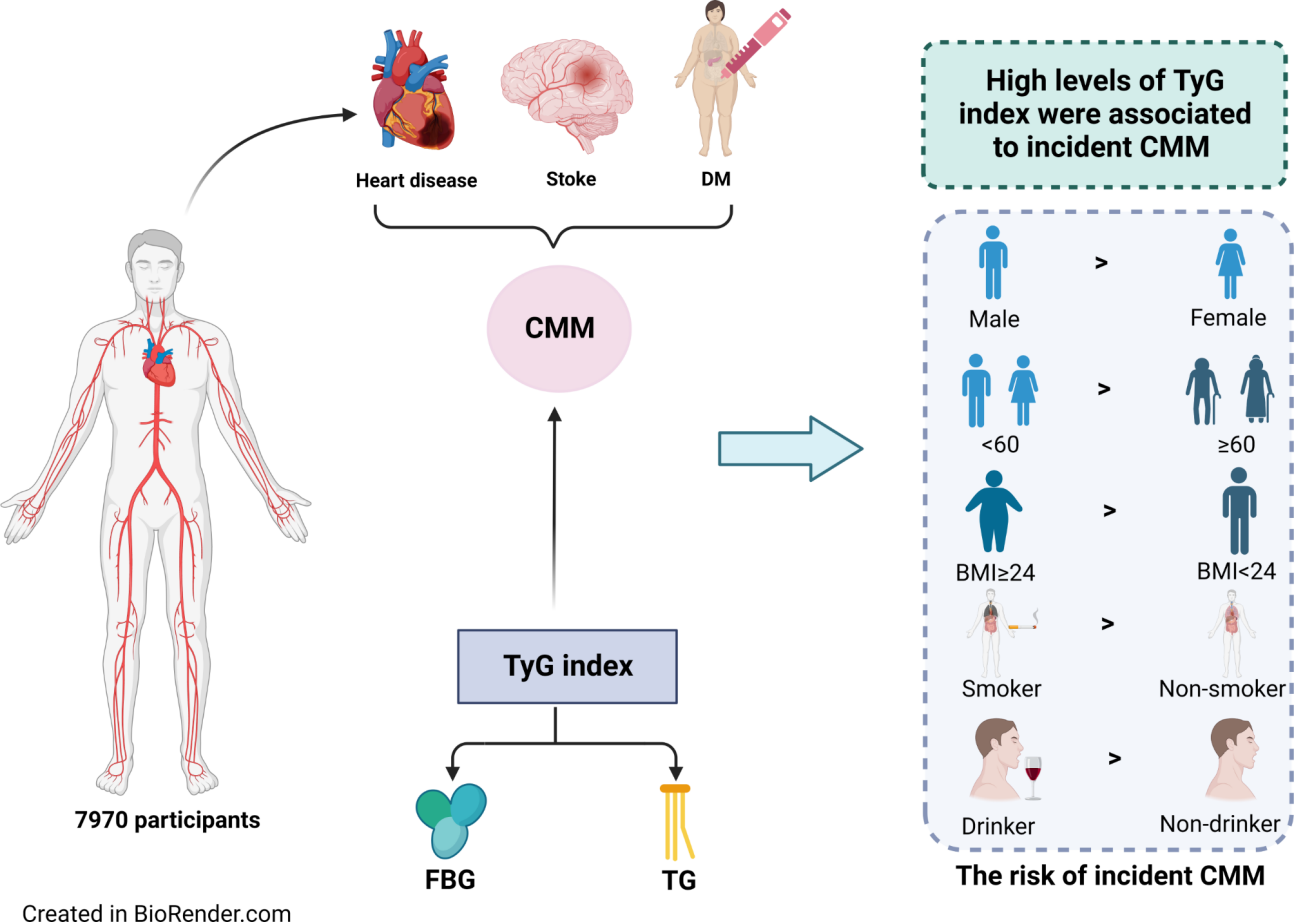

**Supplementary Information:**

The online version contains supplementary material available at 10.1186/s13098-023-01205-8.

## Introduction

Multi-morbidity, which is the simultaneous presence of at least two chronic diseases in an individual, has become an international health challenge and is a global health research priority [1–3]. It imposes substantial health and economic burdens on society and individuals, and is associated with a reduction in quality of life, a reduction in life expectancy, and an increase in healthcare expenditure [4, 5]. Cardiometabolic multi-morbidity (CMM), defined as the presence of at least two of stroke, heart disease, and diabetes mellitus (DM), which are also known as cardiometabolic diseases (CMDs) [6–8], is considered as one of the most serious and common multi-morbidity profiles [9]. CMM has been reported to be associated with a two-fold higher risk of all-cause mortality than an individual CMD [4, 10]. Moreover, a recent study showed that individuals with CMM are at a 1.89-times higher risk of mental stress than those with no cardiometabolic condition [11]. However, despite the increasing burden of disease and concerns about the effects of these diseases on health, previous studies have focused on individual CMDs, rather than on identifying predictors of CMM [12–14].

A great deal of evidence shows that lipid and glucose metabolic disorders play crucial roles in the initiation, progression, and pathogenesis of CMD [15–17], and insulin resistance (IR) underpins these relationships. The hyperinsulinemic-euglycemic clamp is regarded as the gold-standard method of assessing IR [18], but it is difficult to perform in clinical practice because of the expense associated and the complexity of the procedure. Fortunately, several more convenient and valid alternative measures of IR have been established, including the TyG index, which is a highly accurate method of diagnosing IR (sensitivity: 96.5%; specificity: 85.0%) [19].

Previous studies have principally focused on the relationships of TyG index with individual CMDs, such as DM [17, 20], prediabetes [21], adverse cardiovascular events [22, 23], and ischemic stroke [24]. However, to date, few studies have focused on the relationship between IR and CMM. More importantly, the evidence for CMM prevention is more concentrated in European and American populations, and there are also inconsistent results of risk factors for CMM between European, American and Asian populations [6, 7, 25]. For example, overall obesity, quantified by body mass index (BMI) is a crucial risk factor for CMM [6, 7], while abdominal obesity is superior to BMI in predicting CMM in Chinese population [25].

To address these knowledge gaps mentioned above, and considering that the huge health and economic burdens of CMM on society and individuals, we collected data from CHARLS, a prospective, nationwide and representative cohort study, and aimed to improve the current status of the primary prevention of CMM through investigating the association between IR and CMM.

## Methods

### Study design and participants

The study sample comprised participants in the CHARLS (data are available at http://charls.pku.edu.cn/en), which has been described in detail previously [26]. In brief, the CHARLS is a prospective, nationwide cohort study of residents in rural and urban areas of China of ≥ 45 years of age that commenced in 2011 and their spouse [27]. To prepare a representative sample, multi-stage probability sampling of participants in the baseline survey was performed. This survey took place in 2011 and was of individuals resident in 28 provinces on the Chinese mainland [27]. To date, five follow-up surveys have been completed, in 2011, 2013, 2015, 2018, and 2020, but the 2020 data have not been released to the public at the time of writing.

**Fig. 1:**
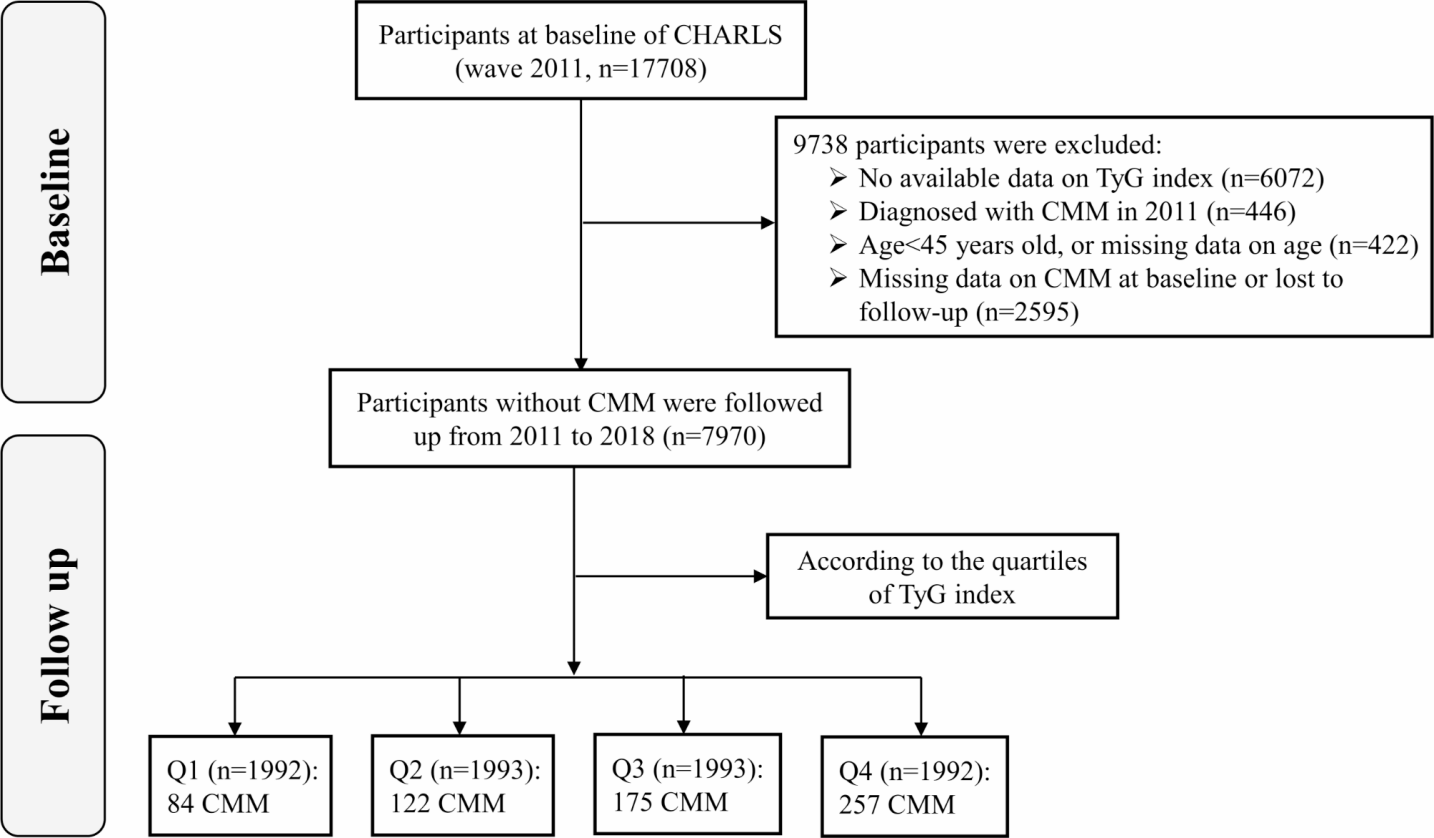
The flowchart of study participants

We initially screened 17,708 participants in the CHARLS and selected 7,970 participants, who were placed into four subgroups according to quartile of TyG index at baseline. The other 9,738 individuals were excluded because of missing laboratory measurements at baseline (n = 6,072), the presence of self-reported confirmed CMM (n = 446), age < 45 years or missing data regarding age (n = 422), missing data/unknown status regarding CMM, or death or loss to follow-up (n = 2,595). The enrolled participants were followed up every 1–2 years until 2018. Fig. 1 details the selection criteria.

### Collection of study data and measurements made

Extensive baseline information was collected by trained interviewers in accordance with standard procedures, including their age, sex (male or female), total cholesterol (TC) concentration, alcohol consumption and smoking status (current, former and never) [28], low-density lipoprotein (LDL) concentration, and region of residence (south or north). After at least 5 min spent sitting down, the blood pressure of the participants was measured three times by a trained interviewer at 45-second intervals using a digital sphygmomanometer (Omron TM HEM-7200). We used a digital weighing scale (Omron Corporation, HN-286) and a stadiometer (Seca Corporation, 213) to measure body mass and height to within 0.1 kg and 0.1 cm, respectively, while the participants were wearing lightweight clothes and no shoes. Body mass index (kg/m^2^) was calculated as body mass/height^2^.

After an overnight fast, three tubes of venous blood were collected from the participants by professional staff, and parameters were measured according to standard procedures. Fasting blood glucose (FBG) and serum lipid parameters were measured using enzymatic colorimetric assays. Blood urea nitrogen (BUN) was measured using an enzymatic UV method involving urease. The serum creatinine and uric acid (UA) concentrations and glycosylated hemoglobin (HbA1c) were measured using the rate-blanked and compensated Jaffe creatinine method, the UA Plus method, and boronate-affinity high-performance liquid chromatography, respectively.

### Definitions

North and south region were defined according to the Qinling Mountains-Huaihe River Line [28]. Hypertension was diagnosed based on a self-reported physician diagnosis (a positive response to “Have you been diagnosed with hypertension?”), and/or recent use of an antihypertensive agent (a positive response to “Are you currently taking any antihypertensive drugs to treat or control your blood pressure?”), and/or a systolic blood pressure/diastolic blood pressure (SBP/DBP) ≥ 140/90 mmHg [29]. Stroke and heart disease were recorded in the presence of a self-reported physician diagnosis (a positive response to “Have you been diagnosed with stroke and/or heart disease?”). DM was defined as follows [30]: FBG ≥ 126 mg/dL, and/or an HbA1c level ≥ 6.5%, and/or a self-reported physician diagnosis (a positive response to “Have you been diagnosed with DM?”), and/or the recent use of a hypoglycemic agent (insulin and/or any hypoglycemic drug). Heart disease and kidney disease were defined as the self-report of physician’s diagnosis (a positive response to “Have you been diagnosed with any heart disease, including angina, congestive heart failure, myocardial infraction, coronary heart disease and any other heart problems ?”) or (a positive response to “Have you been diagnosed with any kidney disease [did not include tumor or cancer]?”), following the previous CHARLS study [31].

### Exposure and endpoint

As in the previous study, the TyG index was calculated as ln(TG (mg/dL) × FBG (mg/dL)/2) [32]. The endpoint was CMM, defined as the simultaneous presence of at least two of the following: stroke, heart disease, and DM [6, 9].

### Handling of missing data

The distribution of missing data for the included participants is shown in Supplementary file, Table S1. Data from participants with a complete set of data were included in the regression models, but an additional analysis was performed using the multiple imputations method (random forest) to impute the missing data and thereby minimize the potential bias.

### Statistical analysis

Statistical analyses were conducted using STATA MP version 17.0 and RStudio 4.2.1. The normal distribution and equality of variance of continuous datasets were evaluated using the Kolmogorov-Smirnov test and Levene test, respectively. All the continuous datasets were normally distributed and are described using mean ± standard deviation; one-way ANOVA was used to identify differences among the groups. Categorical datasets are described as counts and percentages, and the chi-square test was used to identify differences among the groups. The participants were followed from baseline (2011) to the onset of CMM or the most recent survey (2018), whichever came first. The incidence of CMM is stated per 1,000 person-years. Cumulative incidences of CMM were estimated using Kaplan–Meier curves and log-rank test.

Cox regression analyses were used to estimate the hazard ratio (HR) and 95% confidence interval (CI) of the TyG index for CMM. Three models were constructed: Model 1 was a crude model; Model 2 was adjusted for age, sex, SBP, DBP, BMI, and smoking and alcohol consumption status; and Model 3 was adjusted for these variables, plus marital status, educational level, rural vs. urban residence, heart rate, BUN, serum creatinine, UA, hemoglobin, total cholesterol (TC), LDL, and the presence of stroke, heart disease, or DM. We plotted Schoenfeld residuals against time to evaluate the proportional hazards assumption. In addition, multivariable-adjusted restricted cubic splines (RCSs) were used to assess the level-response association of the TyG index with the risk of incident CMM. All the variables included in the models were evaluated for collinearity, and no clear evidence of multicollinearity was detected (the all-variance inflation factor of the included variables was < 10) (Supplementary file, Figure S1).

Subgroup analyses were performed on data stratified according to the age (< 60 or ≥ 60 years), sex (male or female), BMI (< 24 or ≥ 24 kg/m^2^), residence area (rural or urban area), and smoking and alcohol consumption status (never, former and current smoker or alcohol consumers). In addition, given the substantial effect of blood pressure on the incidence of CMM, we placed participants into normotension (< 120/80 mmHg), prehypertension (≥ 120/80 but < 140/90 mm Hg), and hypertension (as defined above) groups and assessed the relationship of TyG index with the risk of CMM in each group [33]. Several sensitivity analyses were also performed to ensure that the findings were robust. In brief, we sequentially excluded participants who had experienced only stroke (n = 110), only had DM (n = 364), or only had heart disease (n = 752), and simultaneously excluded those mentioned above (experienced stroke, or DM, or heart disease (n = 1,226)). To fully explain the observed association, E-value analysis was performed to quantify the exposure-confounder and confounder-outcome relationships [34]. Moreover, an additional analysis was performed to exclude the potential influence of kidney diseases on the observed association. *P* < 0.05 was defined as indicating statistical significance.

## Results

Figure 1 shows the selection criteria used in the present study. Data for 7,970 participants were analyzed according to the quartile of the TyG index: Q1 (≤ 8.22, n = 1,992), Q2 (8.22–8.59, n = 1,993), Q3 (8.59–9.03, n = 1,993), and Q4 (> 9.03, n = 1,992). The characteristics of the participants who were included and excluded are shown in Supplementary file, Table S2. The age, TyG index, DBP, BMI, and region of residence were similar for these two groups, although there were some significant *P*-values. The excluded individuals had higher SBP (130.66 ± 22.14 mmHg vs. 128.53 ± 20.92 mmHg) and prevalence of DM (7.4% vs. 4.6%) and hypertension (60.5% vs. 46.9%) than the included individuals (all *P* < 0.001).

### Baseline characteristics of the participants

The mean age of the included participants was 58.29 ± 8.76 years, and 54.3% were female. Their baseline characteristics, according to the quartile of the TyG index, are shown in Table 1. The mean TyG index was 8.67 ± 0.66, and Supplementary file, Figure S2 shows the frequency distribution of the TyG index in detail. In general, compared with participants in Q1, those in Q2–Q4 were more likely to be women; less likely to live in a rural area (all *P* < 0.05); less likely to have high SBP, DBP, BMI, hemoglobin, FBG, HbA1c, TC, TG, LDL, and UA; more likely to have hypertension and/or DM; and more likely to have low BUN and high-density lipoprotein concentrations (all *P* < 0.001). The proportions of never smokers and never alcohol consumers were also higher in Q2–Q4 than in Q1. There were no significant differences with respect to marital status or educational level among the quartiles. Supplementary file, Table S3 shows the baseline data for the participants, classified according to whether or not they subsequently developed CMM.

**Table 1:** Baseline characteristics of participants stratified by quartiles of TyG index

Characteristics	Overall	Quartiles of TyG index				
		Q1 (≤ 8.22)	Q2 (> 8.22 to 8.59)	Q3 (> 8.59 to 9.03)	Q4 (> 9.03)	*P* value
n	7970	1992	1993	1993	1992	
TyG index	8.67 ± 0.66	7.94 ± 0.25	8.41 ± 0.10	8.79 ± 0.13	9.55 ± 0.51	< 0.001
Age, years	58.29 ± 8.76	58.31 ± 9.11	58.23 ± 8.76	58.70 ± 8.66	57.90 ± 8.47	0.037
Female, n (%)	4331 (54.3)	921 (46.2)	1079 (54.1)	1168 (58.6)	1163 (58.4)	< 0.001
SBP^&^, mmHg	128.53 ± 20.92	124.79 ± 20.66	127.26 ± 20.43	130.01 ± 21.29	132.17 ± 20.57	< 0.001
DBP^&^, mmHg	75.00 ± 12.07	72.79 ± 11.96	74.34 ± 11.86	75.68 ± 12.11	77.26 ± 11.90	< 0.001
Heart rate^&^, rpm	71.94 ± 10.30	70.42 ± 10.06	71.49 ± 10.08	72.41 ± 10.37	73.50 ± 10.45	< 0.001
BMI^&^, kg/m^2^	23.46 ± 3.58	22.08 ± 3.10	22.93 ± 3.40	23.87 ± 3.61	25.01 ± 3.52	< 0.001
Rural residence, n (%)	5355 (67.2)	1421 (71.3)	1392 (69.8)	1308 (65.6)	1234 (61.9)	< 0.001
Region*, n (%)						0.019
**North**	3658 (45.9)	862 (43.3)	912 (45.8)	924 (46.4)	960 (48.2)	
**South**	4312 (54.1)	1130 (56.7)	1081 (54.2)	1069 (53.6)	1032 (51.8)	
Education, n (%)						0.926
**Junior high school and below**	7232 (90.7)	1811 (90.9)	1817 (91.2)	1805 (90.6)	1799 (90.3)	
**Senior high school**	670 (8.4)	165 (8.3)	161 (8.1)	172 (8.6)	172 (8.6)	
**Tertiary**	68 (0.9)	16 (0.8)	15 (0.8)	16 (0.8)	21 (1.1)	
Marital status, n (%)						0.101
**Married and living with spouse**	6767 (84.9)	1671 (83.9)	1682 (84.4)	1690 (84.8)	1724 (86.5)	
**Others**	1203 (15.1)	321 (16.1)	311 (15.6)	303 (15.2)	268 (13.5)	
**Alcohol consumption, n (%)**						< 0.001
**Never**	4689 (58.8)	1090 (54.7)	1148 (57.6)	1242 (62.3)	1209 (60.7)	
**Former**	630 (7.9)	136 (6.8)	186 (9.3)	168 (8.4)	140 (7.0)	
**Current**	2651 (33.3)	766 (38.5)	659 (33.1)	583 (29.3)	643 (32.3)	
Smoking status, n (%)						< 0.001
**Never**	5049 (63.4)	1157 (58.1)	1261 (63.3)	1311 (65.8)	1320 (66.3)	
**Former**	623 (7.8)	159 (8.0)	142 (7.1)	161 (8.1)	161 (8.1)	
**Current**	2298 (28.8)	676 (33.9)	590 (29.6)	521 (26.1)	511 (25.7)	
Hemoglobin, g/dL	14.41 ± 2.21	14.23 ± 2.28	14.36 ± 2.22	14.37 ± 2.13	14.68 ± 2.18	< 0.001
FBG, mg/dL	108.42 ± 33.42	95.15 ± 14.17	100.94 ± 13.95	105.87 ± 17.20	131.71 ± 54.74	< 0.001
HbA1c^&^, %	5.24 ± 0.76	5.07 ± 0.44	5.13 ± 0.49	5.18 ± 0.53	5.57 ± 1.21	< 0.001
TC, mg/dL	193.55 ± 38.66	179.46 ± 33.35	190.52 ± 33.86	196.02 ± 36.83	208.19 ± 44.01	< 0.001
TG, mg/dL	132.88 ± 108.03	61.11 ± 13.82	90.69 ± 14.61	127.92 ± 22.04	251.84 ± 157.17	< 0.001
HDL, mg/dL	51.32 ± 15.43	60.55 ± 15.11	54.78 ± 14.16	49.00 ± 13.13	40.93 ± 11.86	< 0.001
LDL^&^, mg/dL	116.65 ± 34.75	108.96 ± 29.14	119.86 ± 31.14	123.24 ± 33.88	114.51 ± 41.85	< 0.001
BUN^&^, mg/dL	15.74 ± 4.47	16.38 ± 4.76	15.67 ± 4.39	15.37 ± 4.24	15.52 ± 4.39	< 0.001
UA, mg/dL	4.40 ± 1.21	4.22 ± 1.12	4.27 ± 1.16	4.45 ± 1.19	4.69 ± 1.30	< 0.001
Serum creatinine^&^, mg/dL	0.77 ± 0.18	0.77 ± 0.18	0.76 ± 0.17	0.78 ± 0.18	0.79 ± 0.19	< 0.001
Hypertension, n (%)	3738 (46.9)	750 (37.7)	867 (43.5)	982 (49.3)	1139 (57.18)	< 0.001
Kidney disease^&^, n (%)	445 (5.6)	120 (6.0)	124 (6.2)	105 (5.3)	96 (4.8)	0.181
Stroke, n (%)	110 (1.4)	27 (1.4)	33 (1.7)	28 (1.4)	22 (1.1)	0.524
Heart disease, n (%)	752 (9.4)	165 (8.3)	178 (8.9)	207 (10.4)	202 (10.1)	0.073
DM, n (%)	364 (4.6)	32 (1.6)	50 (2.5)	75 (3.8)	207 (10.4)	< 0.001

BMI, body mass index; BUN, blood urea nitrogen; DBP, diastolic blood pressure; DM, diabetes mellitus; FBG, fasting blood glucose; HbA1c, glycosylated hemoglobin A1c; HDL, high density lipoprotein; LDL, low density lipoprotein; Q, quartile; SBP, systolic blood pressure; TC, total cholesterol; TG, triglycerides; TyG, triglyceride-glucose; UA, uric acid

*Region was divided into north (Inner Mongoria, Beijing, Jilin, Tianjin, Shandong, Shanxi, Xinjiang, Hebei, Henan, Gansu, Liaoning, Shaanxi, Qinghai, and Heilongjiang), and south (Shanghai, Yunnan, Sichuan, Anhui, Guangdong, Guangxi, Jiangsu, Jiangxi, Zhejiang, Hubei, Hunan, Fujian, Guizhou, and Chongqing) based on the Qinling Mountains-Huaihe River Line & data for some participants were missing

### Relationship of TyG index with the incidence of CMM

**Fig. 2:**
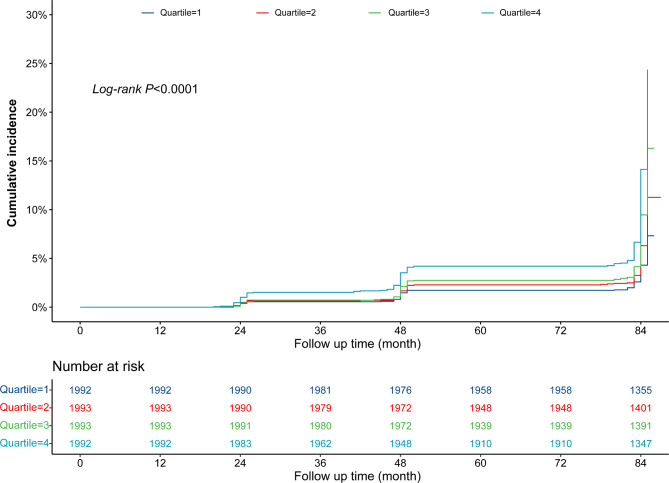
Kaplan–Meier curves for the cumulative incidence of CMM. Quartile 1 is TyG index ≤ 8.22; Quartile 2 is TyG index > 8.22 but ≤ 8.59; Quartile 3 is TyG index > 8.59 but ≤ 9.03; Quartile 4 is TyG index > 9.03. CMM, cardiometabolic multimorbidity; TyG, triglyceride-glucose

**Fig. 3:**
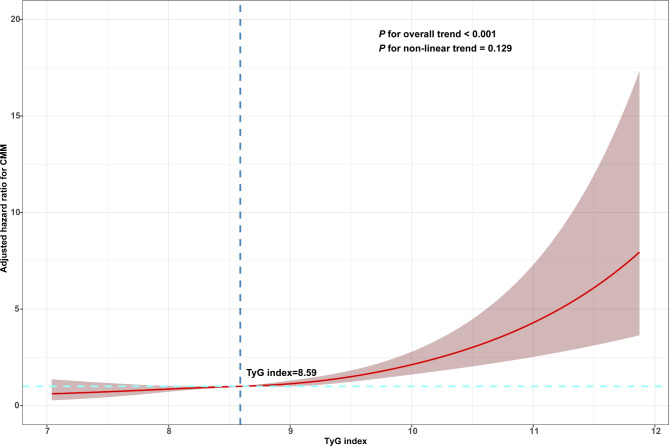
Restricted cubic spline curve for CMM by TyG index after covariate adjustment. Heavy central line represents the estimated adjusted hazard ratio, with shaded ribbons denoting 95% confidence interval. The vertical dotted line indicates the threshold value of TyG index at 8.59. The horizontal dotted line represents the hazard ratio of 1.0. The model is adjusted for age, sex, SBP, DBP, BMI, alcohol consumption and smoking status, marital status, education, rural residence, heart rate, BUN, serum creatinine, UA, hemoglobin, TC, LDL, stroke, heart disease, and DM

In total, 638 (8.01%) incident cases of CMM were documented in the participants. The incidences of CMM were 4.22%, 6.12%, 8.78%, and 12.60% in Q1–Q4, respectively, during a median 84 months of follow-up (interquartile range, 20–87 months). The cumulative incidence of CMM were gradually increased from Q1 to Q4 (Fig. 2). Table 2 shows the incidence and HR with 95% CI of TyG index for CMM. Each 1.0-unit increase in the TyG index was associated with a higher risk of incident CMM in the crude Cox model (HR: 1.76, 95% CI: 1.60–1.94). This relationship was slightly weaker, but remained significant (HR: 1.54, 95% CI: 1.29–1.84) after multivariate adjustment (Model 3) for age, sex, blood pressure, BMI, smoking and alcohol consumption status, marital status, educational status, rural vs. urban residence, heart rate, BUN, serum creatinine, UA, hemoglobin, TC, LDL, and the presence of stroke, heart disease, and DM. Consistent with these results, Model 3 showed that the fully adjusted HRs (95% CIs) of participants in Q3 and Q4 of the TyG index for the development of CMM were 1.41 (1.05–1.90) and 1.61 (1.18–2.20), respectively, vs. those in Q1. Moreover, multivariable-adjusted RCS analysis showed a significant dose-response relationship of TyG index with the risk of CMM (overall *P* < 0.001; non-linear *P* = 0.129) (Fig. 3).

**Table 2:** The association of TyG index with CMM

TyG index	Total N	No. of events (Incident rate^#^)	Model 1		Model 2		Model 3	
			HR (95% CI)	*P* value	HR (95% CI)	*P* value	HR (95% CI)	*P* value
**Continues**								
Per 1.0 increase	7970	638 (11.63)	1.76 (1.60–1.94)	< 0.001	1.51 (1.34–1.69)	< 0.001	1.54 (1.29–1.84)	< 0.001
**Quartiles**								
Q1 (≤ 8.22)	1992	84 (6.17)	Ref.		Ref.		Ref.	
Q2 (> 8.22 to 8.59)	1993	122 (8.87)	1.44 (1.09–1.90)	0.010	1.39 (1.03–1.88)	0.031	1.30 (0.96–1.77)	0.095
Q3 (> 8.59 to 9.03)	1993	175 (12.75)	2.08 (1.60–2.70)	< 0.001	1.57 (1.18–2.10)	0.002	1.41 (1.05–1.90)	0.024
Q4 (> 9.03)	1992	257 (18.91)	3.13 (2.44-4.00)	< 0.001	2.07 (1.56–2.74)	< 0.001	1.61 (1.18–2.20)	0.003
*P* for trend				< 0.001		< 0.001		0.028

Model 1: unadjusted

Model 2: adjusted for age, sex, SBP, DBP, BMI, alcohol consumption and smoking status

Model 3: model 2 + further adjusted for marital status, education, rural residence, heart rate, BUN, serum creatinine, UA, hemoglobin, TC, LDL, stroke, heart disease, and DM

BMI, body mass index; BUN, blood urea nitrogen; CI, confidence interval; CMM, cardiometabolic multimorbidity; DBP, diastolic blood pressure; DM, diabetes mellitus; HR, hazard ratio; LDL, low density lipoprotein; Q, quartile; Ref, reference; SBP, systolic blood pressure; TC, total cholesterol; UA, uric acid

^#^Incident rate was presented as per 1000 person-years of follow-up

The relationship between TyG index and the incidence of CMM, expressed according to blood pressure status, is shown in Table 3. In the participants with prehypertension, the higher quartiles of TyG index were positively associated with the risk of incident CMM (Q3: HR: 1.66, 95% CI: 1.15–5.73, *P* = 0.021; Q4: HR: 2.47, 95% CI: 1.08–5.66, *P* = 0.033) in Model 3, relative to Q1. Consistent with this, each 1.0-unit increment in the TyG index was associated with a 91% increase in the incidence of CMM (HR: 1.91, 95% CI: 1.26–2.90, *P* = 0.002). In addition, increases in TyG index were significantly associated with increases in the incidence of CMM in participants with hypertension (HR: 1.37, 95% CI: 1.10–1.71, *P* = 0.005), but there were no significant differences among the quartiles. Moreover, participants with normotension who were in the higher quartiles of TyG index did not differ from those in Q1 with respect to the risk of CMM.

**Table 3:** The association of TyG index with CMM in different blood pressure status

TyG index	Total N	No. of events (Incident rate^#^)	Model 1		Model 2		Model 3	
			HR (95% CI)	*P* value	HR (95% CI)	*P* value	HR (95% CI)	*P* value
**Normotension**								
TyG index^$^	2367	82 (4.99)	1.67 (1.23–2.25)	0.001	1.46 (1.05–2.04)	0.027	1.47 (0.93–2.30)	0.098
Q1 (≤ 8.22)	765	16 (3.00)	Ref.		Ref.		Ref.	
Q2 (> 8.22 to 8.59)	661	22 (4.80)	1.61 (0.85–3.07)	0.146	1.45 (0.76–2.78)	0.260	1.49 (0.77–2.87)	0.237
Q3 (> 8.59 to 9.03)	532	22 (5.96)	1.95 (1.02–3.71)	0.042	1.57 (0.82–3.03)	0.176	1.66 (0.85–3.22)	0.138
Q4 (> 9.03)	409	22 (7.78)	2.58 (1.36–4.92)	0.004	1.87 (0.96–3.68)	0.067	1.61 (0.84–3.70)	0.135
**Prehypertension**								
TyG index^$^	1865	105 (8.14)	2.06 (1.61–2.64)	< 0.001	1.82 (1.39–2.39)	< 0.001	1.91 (1.26–2.90)	0.002
Q1 (≤ 8.22)	477	9 (2.71)	Ref.		Ref.		Ref.	
Q2 (> 8.22 to 8.59)	465	20 (6.20)	2.31 (1.05–5.07)	0.037	2.02 (0.91–4.45)	0.083	1.79 (0.78–4.16)	0.170
Q3 (> 8.59 to 9.03)	479	35 (10.56)	3.97 (1.91–8.25)	< 0.001	3.21 (1.53–6.74)	0.002	1.66 (1.15–5.73)	0.021
Q4 (> 9.03)	444	41 (13.44)	5.00 (2.43–10.29)	< 0.001	3.61 (1.72–7.55)	0.001	2.47 (1.08–5.66)	0.033
**Hypertension**								
TyG index^$^	3738	451 (17.68)	1.51 (1.35–1.70)	< 0.001	1.38 (1.20–1.59)	< 0.001	1.37 (1.10–1.71)	0.005
Q1 (≤ 8.22)	750	59 (11.50)	Ref.		Ref.		Ref.	
Q2 (> 8.22 to 8.59)	867	80 (13.46)	1.12 (0.80–1.57)	0.509	1.15 (0.79–1.67)	0.479	0.83 (0.57–1.23)	0.352
Q3 (> 8.59 to 9.03)	982	118 (17.56)	1.48 (1.08–2.02)	0.014	1.17 (0.82–1.68)	0.394	0.89 (0.65–1.24)	0.494
Q4 (> 9.03)	1139	194 (25.15)	2.15 (1.61–2.88)	< 0.001	1.59 (1.13–2.24)	0.009	0.91 (0.68–1.21)	0.499

Model 1: unadjusted

Model 2: adjusted for age, sex, SBP, DBP, BMI, alcohol consumption and smoking status

Model 3: model 2 + further adjusted for marital status, education, rural residence, heart rate, BUN, serum creatinine, UA, hemoglobin, TC, LDL, stroke, heart disease, and DM

BMI, body mass index; BUN, blood urea nitrogen; CI, confidence interval; CMM, cardiometabolic multimorbidity; DBP, diastolic blood pressure; DM, diabetes mellitus; HR, hazard ratio; LDL, low density lipoprotein; Q, quartile; Ref, reference; SBP, systolic blood pressure; TC, total cholesterol; UA, uric acid

^#^Incident rate was presented as per 1000 person-years of follow-up

^$^Per 1.0 increase

### Subgroup analyses

**Fig. 4:**
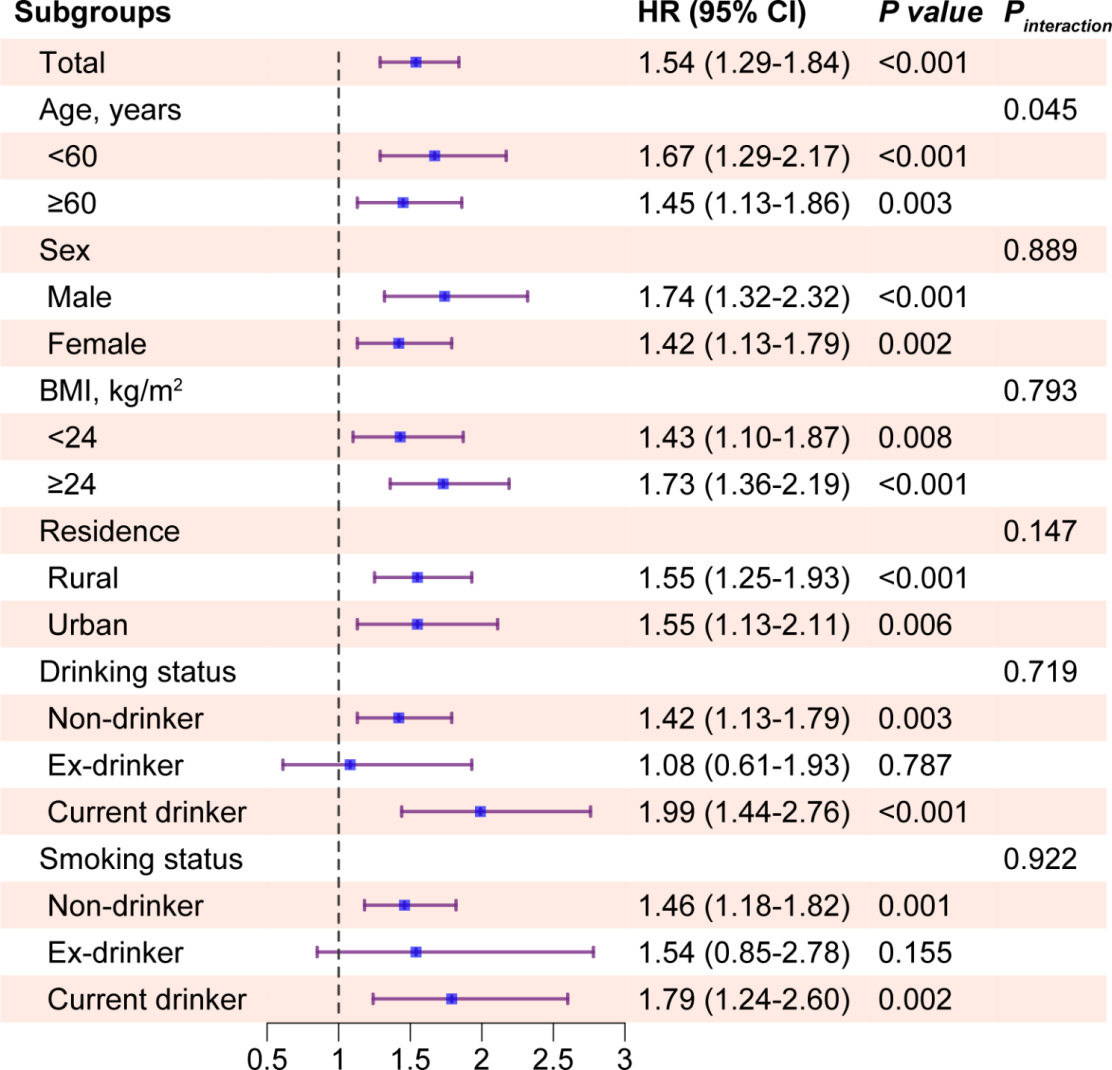
Subgroup and interaction analysis between the TyG index (per 1.0 increase) and CMM across various subgroups

The relationship of TyG index with the incidence of CMM was further assessed in subgroup analyses. Notably, there was an interaction between age and incident CMM (*P* for interaction = 0.045), but both of the subgroups showed close associations with CMM (< 60 years vs. ≥60 years, HR: 1.67, 95% CI 1.29–2.17 and HR: 1.45, 95% CI: 1.13–1.86, respectively). However, the interactions of sex, BMI, residence, alcohol consumption status, smoking status, and the TyG index with the risk of CMM were not significant, and the association was abolished in the former alcohol consumer (HR: 1.08, 95% CI: 0.61–1.93, *P* = 0.787) and former smoker (HR: 1.54, 95% CI: 0.85–2.78, *P* = 0.155) subgroups (Fig. 4).

### Sensitivity analyses

To ensure that these findings were robust, we performed several sensitivity analyses. First, we excluded participants who had DM at baseline (n = 364), and the association of TyG index with CMM persisted (Q3 vs. Q1: HR: 1.50, 95% CI: 1.10–2.06; Q4 vs. Q1: HR: 1.79, 95% CI: 1.28–2.51; for each 1.0-unit increase, HR: 1.72, 95% CI: 1.40–2.11) (Supplementary file, Table S4). We next excluded participants who had experienced stroke at baseline (n = 110), and found no substantial change in the relationship (Q3 vs. Q1: HR: 1.41, 95% CI: 1.03–1.91, *P* = 0.030; Q4 vs. Q1: HR: 1.61, 95% CI: 1.17–2.22, *P* = 0.004; for each 1.0-unit increase, HR: 1.55, 95% CI: 1.29–1.85, *P* < 0.001) (Supplementary file, Table S5). We also found a similar relationship when we excluded participants with heart disease at baseline (n = 752): TyG index correlated with the risk of CMM (HR: 1.49, 95% CI: 1.22–1.82), and the fully adjusted HRs (95% CI) for Q2–Q4 of TyG index for CMM were 1.15 (0.81–1.65), 1.16 (0.82–1.64), and 1.42 (0.99–2.04), respectively, vs. Q1 (Supplementary file, Table S6). Finally, we excluded participants who had experienced stroke or had DM or heart disease at baseline (n = 1,226), and the results were similar to those of the primary analysis (Q4 vs. Q1: HR: 1.62, 95% CI: 1.07–2.44, *P* = 0.022; for each 1.0-unit increase, HR: 1.73, 95% CI: 1.35–2.22, *P* < 0.001) (Table 4).

**Table 4:** The association of TyG index with CMM in participants without stroke, heart disease or DM at baseline

TyG index	Total N	No. of events (Incident rate^#^)	Model 1		Model 2		Model 3	
			HR (95% CI)	*P* value	HR (95% CI)	*P* value	HR (95% CI)	*P* value
*Continues*								
Per 1.0 increase	6744	303 (6.47)	1.71 (1.48–1.98)	< 0.001	1.37 (1.15–1.63)	< 0.001	1.73 (1.35–2.22)	< 0.001
*Quartiles*								
Q1	1687	44 (3.75)	Ref.		Ref.		Ref.	
Q2	1687	60 (5.13)	1.30 (0.89–1.91)	0.175	1.11 (0.74–1.66)	0.604	1.08 (0.72–1.62)	0.712
Q3	1684	84 (7.18)	1.92 (1.35–2.74)	< 0.001	1.31 (0.89–1.92)	0.165	1.21 (0.82–1.79)	0.341
Q4	1686	115 (9.85)	2.69 (1.91–3.78)	< 0.001	1.59 (1.09–2.31)	0.016	1.62 (1.07–2.44)	0.022

Model 1: unadjusted

Model 2: adjusted for age, sex, SBP, DBP, BMI, alcohol consumption and smoking status

Model 3: model 2 + further adjusted for marital status, education, rural residence, heart rate, BUN, serum creatinine, UA, hemoglobin, TC, LDL

BMI, body mass index; BUN, blood urea nitrogen; CI, confidence interval; CMM, cardiometabolic multimorbidity; DBP, diastolic blood pressure; DM, diabetes mellitus; HR, hazard ratio; LDL, low density lipoprotein; Q, quartile; Ref, reference; SBP, systolic blood pressure; TC, total cholesterol; UA, uric acid

^#^Incident rate was presented as per 1000 person-years of follow-up

### Additional analysis

As shown in Supplementary file, Figure S3, we calculated an HR and lower confidence limit of 2.45 and 1.90, respectively, in the E-value analysis for the risk of CMM. The results of the additional analysis were consistent with those of the primary analysis, even after the exclusion of participants with kidney disease (Supplementary file, Table S7) or following the use of multiple imputation to deal with missing data (Supplementary file, Table S8).

## Discussion

In the present cohort study of 7,970 members of the general population in China, we have shown that the risk of incident CMM increases as the TyG index increases, and in particular in individuals with prehypertension, even after adjustment for potential confounders, and this relationship remained in sensitivity and subgroup analyses. To our knowledge, this is the first study to show that the TyG index is a predictor of CMM, independent of age, sex, and BMI. Most importantly, we have identified a novel means of aiding the prevention of CMM, which may have far-reaching beneficial effects on public health and in clinical practice.

CMM, defined as the coexistence of at least two of stroke, DM, and heart disease [6–8], is a common form of multi-morbidity that has been shown to be associated with a higher risk of mortality than each CMD on its own [4]. However, little is known regarding the risk factors for CMM. In recent years, studies aimed at identifying potential risk factors for CMM have been attracting attention. A study of 8,270 participants in the Whitehall II cohort study showed that an unhealthy lifestyle is associated with a 209% higher risk of CMM [7], but accurately quantifying the risk of CMM is very difficult.

A study of data from the UK Biobank showed that exposure to air pollution plays a major role in both the initiation and progression of CMM [35], which is consistent with the findings of another study conducted in China [36]. However, it is very difficult for people to avoid breathing in polluted air. More recently, a study conducted in China explored the relationship of obesity with the risk of CMM [25], but its cross-sectional design meant that causal links could not be confirmed. More importantly, the results obtained concerning the predictive value of BMI for CMM have not been consistent: a pooled analysis of 16 cohort studies conducted in Europe and the United States showed that the risk of CMM increases as BMI increases, and this relationship remained in all the subgroup and sensitivity analyses [6], whereas data collected in China showed that BMI is not an ideal predictor of CMM [25]. These contradictory findings suggest that BMI or obesity might not be a useful predictor of CMM in all populations.

In contrast, a large body of evidence demonstrates the predictive value of IR for incident CMM [37, 38]. IR, which is defined as a failure of insulin to have its physiological effects in target tissues, has been shown not only to contribute the development of atherosclerosis and coronary artery disease in individuals with or without DM, but also to possibly represent a novel and promising predictor of adverse cardiovascular events [37, 39, 40]. Therefore, the early recognition of IR should have important clinical implications, permitting the institution of timely measures aimed at the primary and secondary prevention of cardiovascular events through the risk stratification of patients. However, to date, the relationship between IR and CMM have not been well elucidated.

As mentioned above, the TyG index, which is a novel and validated index of IR [19], is closely associated with individual CMDs. In a retrospective observational study of 5.6 million individuals, Hong et al. found that those in Q4 of the TyG index were at a 26% higher risk of stroke (HR: 1.26, 95% CI: 1.23–1.29), a 31% higher risk of myocardial infarction (HR: 1.31, 95% CI 1.28–1.35), and a 28% higher risk of both (HR: 1.28, 95% CI: 1.26–1.30) [41], which is consistent with the results of the present study. Mounting evidence suggests that the TyG index closely correlates with the prevalence and burden of cerebrovascular disease, and has been proposed to be a useful biomarker for stroke or intracerebral hemorrhage [42, 43]. Data from a study of 12,326 Asian participants showed that the TyG index might be a useful predictor of heart disease [44]. In addition, Zou et al. showed that the TyG index is associated with the prevalence of adverse cardio-cerebrovascular events in women (odds ratio (OR): 1.68, 95% CI: 1.12–2.54), but not in men (OR: 0.95, 95% CI: 0.74–1.21); however, this study involved only a 29.8-month follow-up period [45], which may have biased the findings toward the null hypothesis because there may not have been sufficient time for adverse events to occur in response to high baseline TyG index. More importantly, previous studies reported that TyG index had a better sensitivity and specificity in predicting IR using the hyperglycemic clamp test compared with Homeostasis Model Assessment of Insulin Resistance (HOMA-IR) [46, 47]. Multiple studies have shown IR is a strong predictor of CMD [17, 20, 23]. Elevated HOMA-IR has a significant association with risk of incident cardiovascular events, ischemic stroke and diabetes [48, 49]. We also observed a similar results that higher insulin resistance determined by the TyG index was significantly associated with higher risk of future CMM events. However, a recent study found that the HOMA-IR, was not associated with cardiovascular events in patients with diabetes and ACS who do not receive insulin treatment [50]. Compared with the HOMA-IR, the TyG index does not require the concentration of insulin and may be available all of the patients treated with insulin.

In the present study, a high TyG index was found to be associated with a higher incidence of CMM, independent of conventional risk factors, including TC, age, sex, and LDL. Moreover, we identified a linear correlation of the TyG index with the incidence of CMM. This is in contrast to the findings of a published 10-year follow-up study of 5,014 participants that showed that a high TyG index is not associated with a higher risk of cerebrovascular disease (HR: 1.45, 95% CI: 0.83–2.54) [51]. Similarly, Cho et al. found that individuals with DM in the higher quartiles of TyG index were not more likely to have ischemic heart disease than those in Q1 (all *P* > 0.05) [52]. These inconsistencies in findings may be explained by heterogeneity in the study participants, the sample sizes, the durations of follow-up, and the study designs [51, 52]. In addition, the endpoint in our study is defined as CMM, a composite of DM, stroke and heart disease, which may partly explain the discrepancy with previous studies [51, 52]. More importantly, we enrolled participants from CHARLS, a prospective, nationwide cohort study, rather than including inpatients or outpatients who usually have more underlying diseases, which significantly differs from the two studies mentioned above [51, 52]. Moreover, several recent systematic review and meta-analysis suggested that elevated TyG index was significantly associated with higher risk of hypertension, arterial stiffness, CVD and heart failure [53–56], which is similar with our findings.

Previous studies have principally evaluated the utility of the TyG index for patients developing only one CMD. Here, we have explored the potential for the use of the TyG index to predict the risk of incident CMM. Our findings remained significant even after adjustment for conventional confounders, such as SBP, DBP, BMI, and the prevalence of a single CMD. In the subgroup analyses, the interactions were not significant, except with respect to age and the risk of CMM, but both of the two subgroups were closely associated with the incidence of CMM, which suggests that the findings of the present study may be applicable to the general population.

In addition, individuals with prehypertension and values of the TyG index in the higher quartiles were more likely to develop CMM than those in Q1, although the relationship was blunted when the participants were categorized according to whether they had normotension or hypertension, suggesting that individuals with prehypertension are at a higher risk of CMM and that physicians should pay more attention to these individuals in clinical practice. Similarly, the results of previous studies have suggested that individuals with prehypertension are at a higher risk of adverse cardiovascular events [57, 58], which may be because of the lack of attention paid to prehypertension and delays to appropriate interventions because the current international guidelines do not provide a consensus regarding the treatment of this group of patients [29, 59]. Therefore, further prospective intervention studies are urgently needed to assess the effects of prehypertension on the subsequent risk of CMM and to determine the optimal time for interventions to be made. Finally, the additional and sensitivity analyses showed that the associations identified in the primary analysis were robust. Notably, the E-value analysis suggested that the effects of unmeasured confounding factors were minor, further confirming that the present findings are reliable.

The mechanisms underpinning the association of TyG index with CMM have not been characterized, but may involve the following. First, IR is involved in the pathogenesis of atherosclerosis: high circulating insulin concentrations cause a reduction in the production of nitric oxide *via* the activation of serum and glucocorticoid kinase 1, and the lower nitric oxide concentration, in turn, leads to matrix protein deposition and fibrosis [60]. Second, the lipid and glucose metabolic disorders that are induced by IR may cause the overproduction of reactive oxide species through the activation of signaling pathways, including the protein kinase C pathway and the nuclear factor (NF)­κB pathway, which may incite CMM [36]. Third, IR causes fluid retention and high blood pressure *via* the ectopic synthesis of angiotensinogen [61] and the consequent inappropriate activation of the RAAS [62]. Finally, insulin promotes thrombosis and platelet aggregation by impairing fibrinolysis, secondary to an increase in the circulating concentration of plasminogen activator inhibitor 1 [63].

The prospective design and nationwide nature of the present cohort study, as well as its large sample size, permitted us to identify potential risk factors for CMM in a robust fashion. The present study is the first to evaluate the predictive value of the TyG index for CMM using a prospective design and in a nationwide cohort with long-term follow-up. We have addressed the knowledge gap regarding the relationship between IR and CMM, which should aid the prevention of CMM by permitting better risk stratification of patients. However, this study had several limitations. First, although many potential confounding factors have been adjusted for, the possibility of residual confounding cannot be completely eliminated. However, the E-value analysis indicated that there was only minor unmeasured confounding and the observed association remained in our subgroup and sensitivity analyses. Second, CMM was defined using a self-reported physician diagnosis, which may have led to information bias. Therefore, large-scale, randomized controlled trials are urgently needed to confirm the present findings. Third, we only enrolled individuals aged ≥ 45 years in the study, which may limit the generalizability of the results. Finally, because it was an observational study, we only included data from China and we excluded a large number of participants, although this is common in cohort studies of this kind, suggesting some selection bias is inevitable. Therefore, although the association remained in all the subgroup and sensitivity analyses, the findings should be interpreted and generalized with caution. Further studies of people of other ethnicities and in other countries should thus be performed to test the generalizability of the findings.

## Conclusions

In the present study, we have shown that a high TyG index is positively associated with a higher risk of incident CMM. Therefore, physicians may be able to reduce the risk of CMM in their patients by keeping their TyG indexes low. Because the TyG index is a readily available surrogate index of IR, early and close monitoring could be used to identify patients at high risk of CMM, thereby significantly improving their prognosis, which may exert far-reaching significance on public health.

### Electronic supplementary material

Below is the link to the electronic supplementary material.

Supplementary Material 1

## Data Availability

The data supporting the findings of this study are available the CHARLS website (http://charls.pku.edu.cn/en).

## List of abbreviations

IR: insulin resistance
BUN: blood urea nitrogen
CI: confidence interval
CMD: cardiometabolic diseases
CMM: cardiometabolic multimorbidity
DBP: diastolic blood pressure
OR: odds ratio
HbA1c: glycosylated hemoglobin
DM: diabetes mellitus
FBG: Fasting blood glucose
HR: hazard ratio
Q: quartile
RCS: restricted cubic splines
SBP: systolic blood pressure
TyG: triglyceride-glucose
BMI: body mass index
UA: uric acid
