# Supplementary material for: Relationship of triglyceride-glucose index with cardiometabolic multi-morbidity in China: evidence from a national survey
Source: Diabetol Metab Syndr. 2023 Nov 6;15:226. doi: 10.1186/s13098-023-01205-8 (PMC10626797; doi:10.1186/s13098-023-01205-8)
Supplement: Supplementary file 1 — Supplementary Material 1 [file 13098_2023_1205_MOESM1_ESM.docx]

**Supplementary file Content**

**Supplementary Figures**

**Figure S1.** The variance inflation factor (VIF) values for all variables in our model 3.

**Abbreviations**: BMI, body mass index; BUN, blood urea nitrogen; DBP, diastolic blood pressure; DM, diabetes mellitus; LDL, low density lipoprotein; SBP, systolic blood pressure; TC, total cholesterol; TyG, triglyceride-glucose; UA, uric acid

**Model 3**: adjusted for age, sex, SBP, DBP, BMI, alcohol consumption and smoking status, marital status, education, rural residence, heart rate, BUN, serum creatinine, UA, hemoglobin, TC, LDL, stroke, heart disease, and DM

**Figure S2.** Distribution of TyG index in the study participants.

**Figure S3.** E-value analysis to evaluate the extent of unmeasured confounders that would be required to negate the observed results.

**Supplementary Tables**

**Table S1.** Distribution of missing data.

**Table S2.** Baseline characteristics of excluded and included participants.

**Table S3.** Baseline characteristics of participants stratified by outcome.

**Table S4.** The association of TyG index with CMM after excluding individuals with DM.

**Table S5.** The association of TyG index with CMM after excluding individuals with stroke.

**Table S6.** The association of TyG index with CMM after excluding individuals with heart disease.

**Table S7.** The association of TyG index with CMM after excluding individuals with kidney disease.

**Table S8.** The association of TyG index with CMM after imputing the baseline missing values.


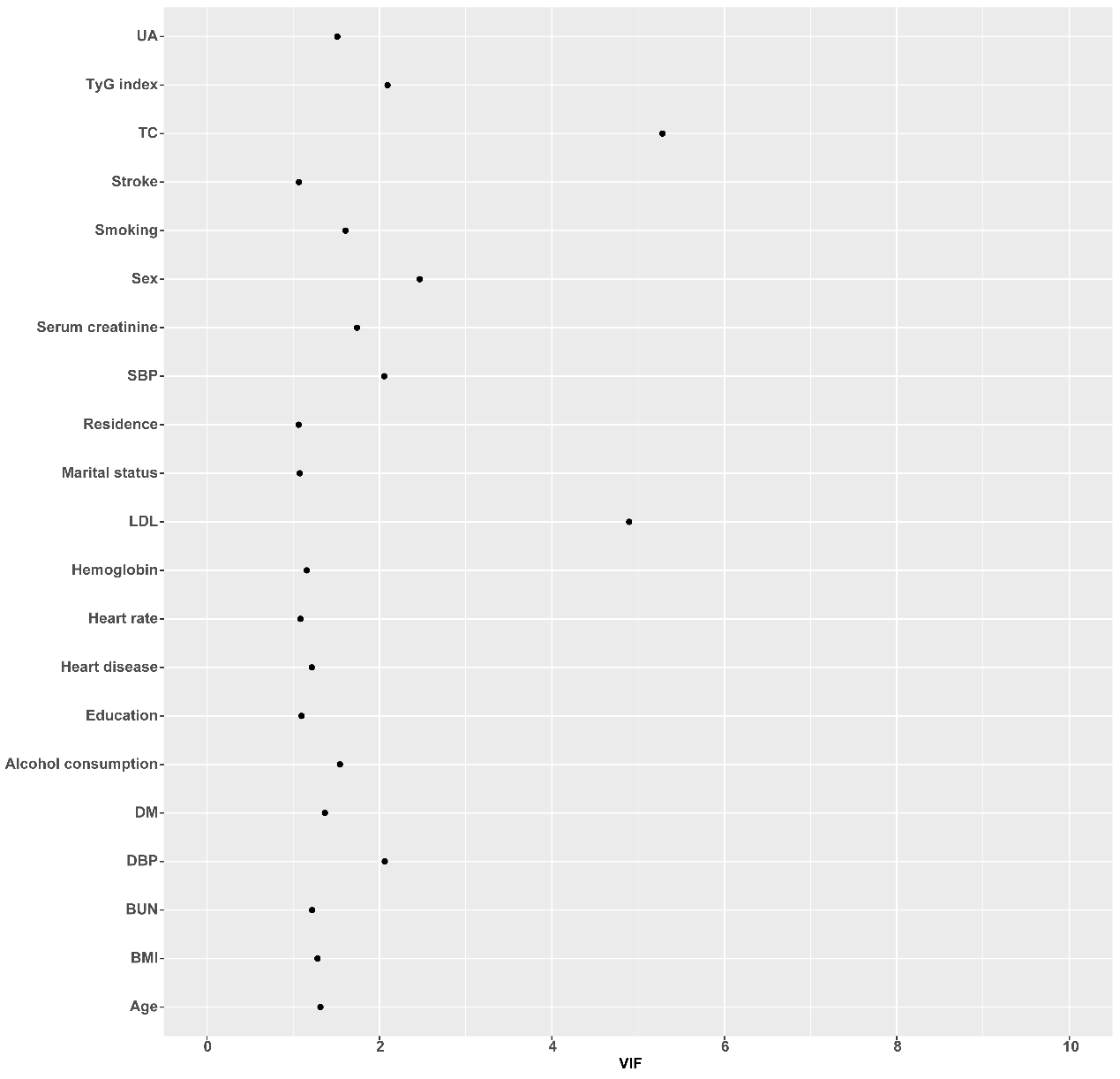


**Figure S1.** The variance inflation factor (VIF) values for all variables in our model 3.

**Abbreviations**: BMI, body mass index; BUN, blood urea nitrogen; DBP, diastolic blood pressure; DM, diabetes mellitus; LDL, low density lipoprotein; SBP, systolic blood pressure; TC, total cholesterol; TyG, triglyceride-glucose; UA, uric acid

**Model 3**: adjusted for age, sex, SBP, DBP, BMI, alcohol consumption and smoking status, marital status, education, rural residence, heart rate, BUN, serum creatinine, UA, hemoglobin, TC, LDL, stroke, heart disease, and DM


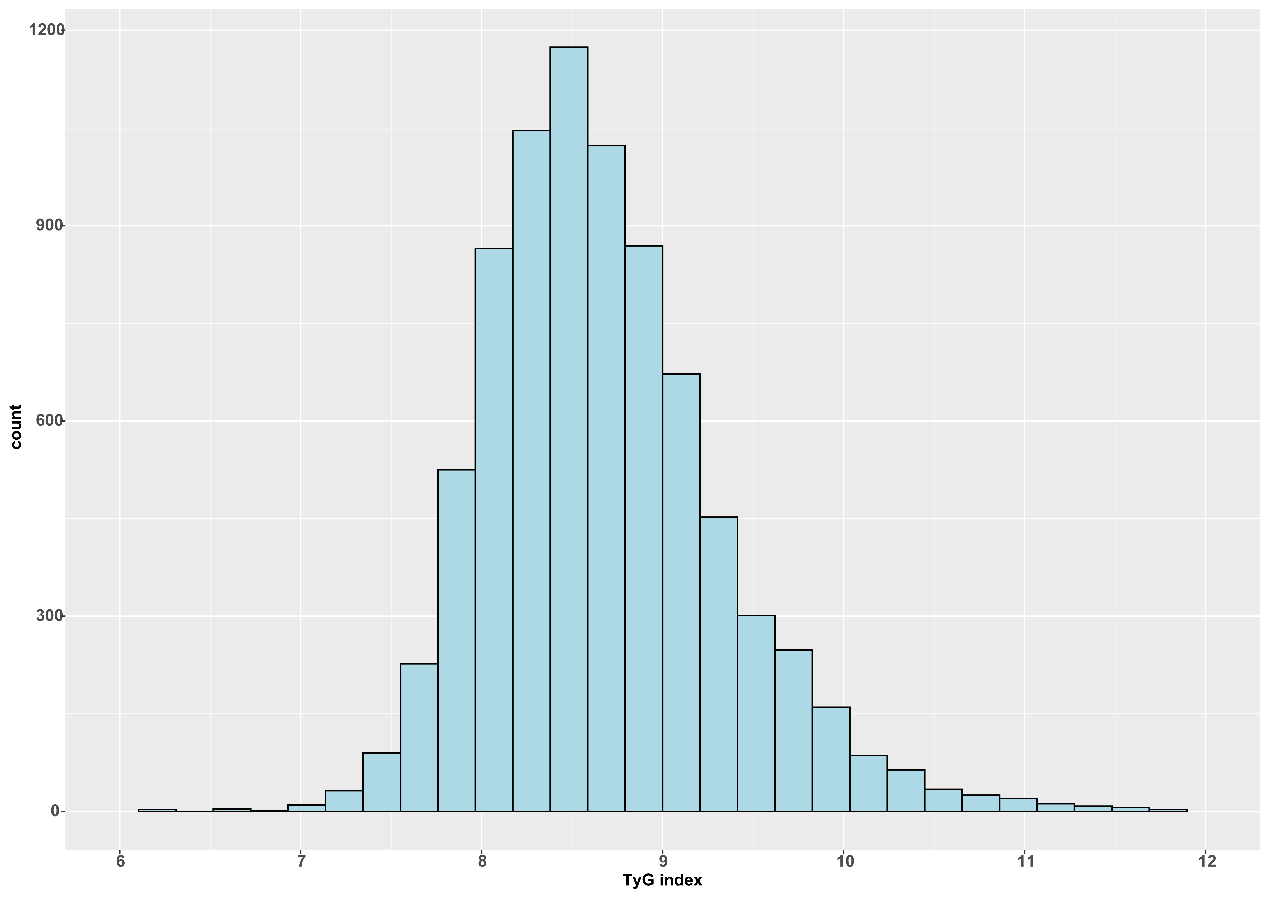


**Figure S2.** Distribution of TyG index in the study participants.


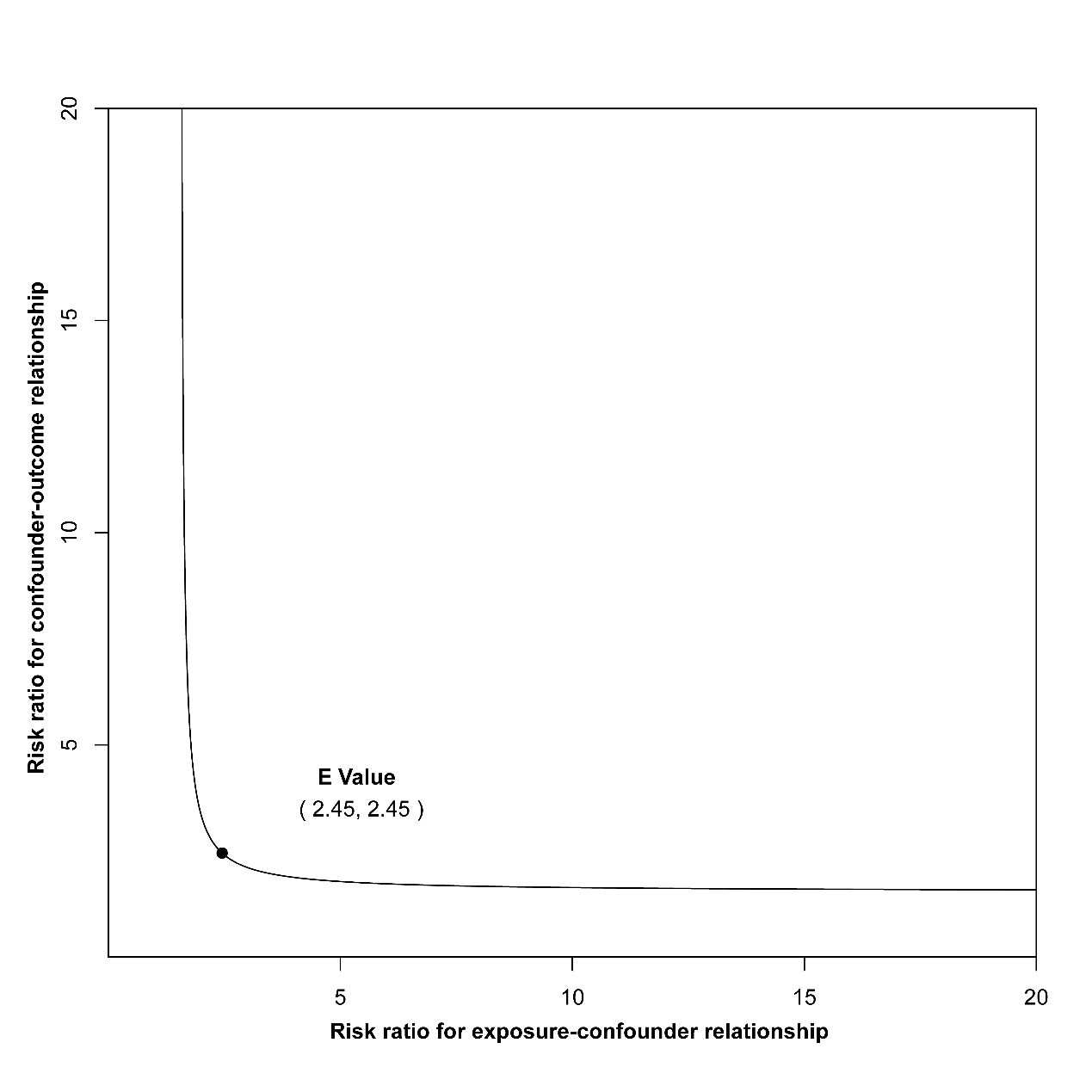


**Figure S3.** E-value analysis to evaluate the extent of unmeasured confounders that would be required to negate the observed results.

**Table S1.** Distribution of missing data.

| **Characteristics** | **No. of missing values** | **Percent(%)** | **Disposition** |
| --- | --- | --- | --- |
| LDL | 13 | 0.16 | Multiple imputation |
| Serum creatinine | 3 | 0.04 | Multiple imputation |
| BUN | 1 | 0.01 | Multiple imputation |
| HbA1c | 168 | 2.11 | Multiple imputation |
| Kidney disease | 22 | 0.28 | Multiple imputation |
| SBP | 197 | 2.47 | Multiple imputation |
| DBP | 199 | 2.50 | Multiple imputation |
| Heart rate | 200 | 2.51 | Multiple imputation |
| BMI | 313 | 3.92 | Multiple imputation |
| Total | 1116 | 14.00 | Multiple imputation |

BMI, body mass index; BUN, blood urea nitrogen; DBP, diastolic blood pressure; HbA1c, glycosylated hemoglobin A1c; LDL, low density lipoprotein; SBP, systolic blood pressure

**Table S2.** Baseline characteristics of excluded and included participants.

| **Characteristics** | **Overall** | **Excluded** | **Included** | ***P* value** |
| --- | --- | --- | --- | --- |
| n | 17708 | 9738 | 7970 |  |
| TyG index | 8.70 ± 0.68 | 8.75 ± 0.72 | 8.67 ± 0.66 | <0.001 |
| Age, years | 58.50 ± 10.17 | 58.67 ± 11.21 | 58.29 ± 8.76 | 0.013 |
| Female, n (%) | 9228 (52.1) | 4897 (50.3) | 4331 (54.3) | <0.001 |
| SBP, mmHg | 129.58 ± 21.56 | 130.66 ± 22.14 | 128.53 ± 20.92 | <0.001 |
| DBP, mmHg | 75.48 ± 12.26 | 75.98 ± 12.43 | 75.00 ± 12.07 | <0.001 |
| Heart rate, rpm | 72.35 ± 10.56 | 72.76 ± 10.81 | 71.94 ± 10.30 | <0.001 |
| Rural residence, n (%) | 10537 (59.5) | 5182 (53.2) | 5355 (67.2) | <0.001 |
| Region* , n (%) |  |  |  | <0.001 |
| North | 7841 (44.3) | 4183 (43.0) | 3658 (45.9) |  |
| South | 9867 (55.7) | 5555 (57.0) | 4312 (54.1) |  |
| Education, n (%) |  |  |  | <0.001 |
| Junior high school and below | 15545 (87.9) | 8313 (85.5) | 7232 (90.7) |  |
| Senior high school | 1793 (10.1) | 1123 (11.6) | 670 (8.4) |  |
| Tertiary | 354 (2.0) | 286 (2.9) | 68 (0.9) |  |
| Marital status, n (%) |  |  |  | <0.001 |
| Married and living with spouse | 14170 (80.0) | 7403 (76.0) | 6767 (84.9) |  |
| Others | 3538 (20.0) | 2335 (24.0) | 1203 (15.1) |  |
| Alcohol consumption, n (%) |  |  |  | 0.157 |
| Never | 10498 (59.3) | 5809 (59.7) | 4689 (58.8) |  |
| Former | 1443 (8.1) | 813 (8.3) | 630 (7.9) |  |
| Current | 5767 (32.6) | 3116 (32.0) | 2651 (33.3) |  |
| Smoking status, n (%) |  |  |  | 0.002 |
| Never | 11420 (64.5) | 6371 (65.4) | 5049 (63.4) |  |
| Former | 1417 (8.0) | 794 (8.2) | 623 (7.8) |  |
| Current | 4871 (27.5) | 2573 (26.4) | 2298 (28.8) |  |
| Hemoglobin, g/dL | 14.38 ± 2.22 | 14.33 ± 2.25 | 14.41 ± 2.21 | 0.091 |
| FBG, mg/dL | 110.30 ± 37.33 | 114.40 ± 44.41 | 108.42 ± 33.42 | <0.001 |
| HbA1c, % | 5.26 ± 0.82 | 5.32 ± 0.94 | 5.24 ± 0.76 | <0.001 |
| TC, mg/dL | 192.97 ± 38.89 | 191.71 ± 39.37 | 193.55 ± 38.66 | 0.017 |
| TG, mg/dl | 134.91 ± 110.26 | 139.28 ± 114.83 | 132.88 ± 108.03 | 0.004 |
| HDL, mg/dL | 50.84 ± 15.33 | 49.82 ± 15.05 | 51.32 ± 15.43 | <0.001 |
| LDL, mg/dL | 115.99 ± 34.91 | 114.58 ± 35.21 | 116.65 ± 34.75 | 0.003 |
| BUN, mg/dL | 15.75 ± 4.65 | 15.78 ± 5.02 | 15.74 ± 4.47 | 0.677 |
| UA, mg/dL | 4.46 ± 1.27 | 4.60 ± 1.38 | 4.40 ± 1.21 | <0.001 |
| Serum creatinine, mg/dL | 0.78 ± 0.24 | 0.81 ± 0.32 | 0.77 ± 0.18 | <0.001 |
| Hypertension, n (%) | 9698 (54.3) | 5860 (60.5) | 3738 (46.9) | <0.001 |
| Kidney disease, n (%) | 979 (5.6) | 534 (5.6) | 445 (5.6) | 0.955 |
| Stroke, n (%) | 486 (2.8) | 376 (3.9) | 110 (1.4) | <0.001 |
| Heart disease, n (%) | 2130 (12.2) | 1378 (14.5) | 752 (9.4) | <0.001 |
| DM, n (%) | 1062 (6.1) | 698 (7.4) | 364 (4.6) | <0.001 |

BUN, blood urea nitrogen; DBP, diastolic blood pressure; DM, diabetes mellitus; FBG, fasting blood glucose; HbA1c, glycosylated hemoglobin A1c; HDL, high density lipoprotein; LDL, low density lipoprotein; Q, quartile; SBP, systolic blood pressure; TC, total cholesterol; TG, triglycerides; TyG, triglyceride-glucose; UA, uric acid

*Region was divided into north (Inner Mongoria, Beijing, Jilin, Tianjin, Shandong, Shanxi, Xinjiang, Hebei, Henan, Gansu, Liaoning, Shaanxi, Qinghai, and Heilongjiang), and south (Shanghai, Yunnan, Sichuan, Anhui, Guangdong, Guangxi, Jiangsu, Jiangxi, Zhejiang, Hubei, Hunan, Fujian, Guizhou, and Chongqing) based on the Qinling Mountains-Huaihe River Line

**Table S3.** Baseline characteristics of participants stratified by outcome.

| **Characteristics** | **Overall** | **Without CMM** | **With CMM** | ***P* value** |
| --- | --- | --- | --- | --- |
| n | 7970 | 7332 | 638 |  |
| TyG index | 8.67 ± 0.66 | 8.65 ± 0.65 | 8.97 ± 0.75 | <0.001 |
| Age, years | 58.29 ± 8.76 | 58.13 ± 8.77 | 60.06 ± 8.47 | <0.001 |
| Female, n (%) | 4331 (54.3) | 3945 (53.8) | 386 (60.5) | 0.001 |
| SBP, mmHg | 128.53 ± 20.92 | 127.78 ± 20.58 | 137.18 ± 22.79 | <0.001 |
| DBP, mmHg | 75.00 ± 12.07 | 74.69 ± 11.99 | 78.60 ± 12.37 | <0.001 |
| Heart rate, rpm | 71.94 ± 10.30 | 71.83 ± 10.25 | 73.21 ± 10.82 | 0.003 |
| BMI, kg/m^2^ | 23.46 ± 3.58 | 23.31 ± 3.51 | 25.26 ± 3.85 | <0.001 |
| Rural residence, n (%) | 5355 (67.2) | 4966 (67.7) | 389 (61.0) | <0.001 |
| Region* , n (%) |  |  |  | <0.001 |
| North | 3658 (45.9) | 3247 (44.3) | 411 (64.4) |  |
| South | 4312 (54.1) | 4085 (55.7) | 227 (35.6) |  |
| Education, n (%) |  |  |  | 0.684 |
| Junior high school and below | 7232 (90.7) | 6658 (90.8) | 574 (90.0) |  |
| Senior high school | 670 (8.4) | 613 (8.4) | 57 (8.9) |  |
| Tertiary | 68 (0.9) | 61 (0.8) | 7 (1.1) |  |
| Marital status, n (%) |  |  |  | 0.972 |
| Married and living with spouse | 6767 (84.9) | 6225 (84.9) | 542 (85.0) |  |
| Others | 1203 (15.1) | 1107 (15.1) | 96 (15.0) |  |
| Alcohol consumption, n (%) |  |  |  | <0.001 |
| Never | 4689 (58.8) | 4300 (58.6) | 389 (61.0) |  |
| Former | 630 (7.9) | 559 (7.6) | 71 (11.1) |  |
| Current | 2651 (33.3) | 2473 (33.7) | 178 (27.9) |  |
| Smoking status, n (%) |  |  |  | <0.001 |
| Never | 5049 (63.4) | 4618 (63.0) | 431 (67.6) |  |
| Former | 623 (7.8) | 556 (7.6) | 67 (10.5) |  |
| Current | 2298 (28.8) | 2158 (29.4) | 140 (21.9) |  |
| Hemoglobin, g/dL | 14.41 ± 2.21 | 14.39 ± 2.20 | 14.63 ± 2.28 | 0.008 |
| FBG, mg/dL | 108.42 ± 33.42 | 107.01 ± 30.43 | 124.61 ± 55.04 | <0.001 |
| HbA1c, % | 5.24 ± 0.76 | 5.20 ± 0.69 | 5.65 ± 1.27 | <0.001 |
| TC, mg/dL | 193.55 ± 38.66 | 193.02 ± 38.48 | 199.58 ± 40.17 | <0.001 |
| TG, mg/dl | 132.88 ± 103.03 | 130.53 ± 106.11 | 159.89 ± 124.99 | <0.001 |
| HDL, mg/dL | 51.32 ± 15.43 | 51.69 ± 15.44 | 47.04 ± 14.72 | <0.001 |
| LDL, mg/dL | 116.65 ± 34.75 | 116.23 ± 34.39 | 121.44 ± 38.38 | <0.001 |
| BUN, mg/dL | 15.74 ± 4.47 | 15.76 ± 4.47 | 15.47 ± 4.42 | 0.123 |
| UA, mg/dL | 4.40 ± 1.21 | 4.39 ± 1.20 | 4.52 ± 1.30 | 0.009 |
| Serum creatinine, mg/dL | 0.77 ± 0.18 | 0.77 ± 0.18 | 0.79 ± 0.20 | 0.007 |
| Hypertension, n (%) | 3738 (46.9) | 3287 (44.8) | 451 (70.7) | <0.001 |
| Kidney disease, n (%) | 445 (5.6) | 377 (5.2) | 68 (10.7) | <0.001 |
| Stroke, n (%) | 110 (1.4) | 82 (1.1) | 28 (4.4) | <0.001 |
| Heart disease, n (%) | 752 (9.4) | 565 (7.7) | 187 (29.3) | <0.001 |
| DM, n (%) | 364 (4.6) | 244 (3.3) | 120 (18.8) | <0.001 |

BMI, body mass index; BUN, blood urea nitrogen; DBP, diastolic blood pressure; DM, diabetes mellitus; FBG, fasting blood glucose; HbA1c, glycosylated hemoglobin A1c; HDL, high density lipoprotein; LDL, low density lipoprotein; Q, quartile; SBP, systolic blood pressure; TC, total cholesterol; TG, triglycerides; TyG, triglyceride-glucose; UA, uric acid

*Region was divided into north (Inner Mongoria, Beijing, Jilin, Tianjin, Shandong, Shanxi, Xinjiang, Hebei, Henan, Gansu, Liaoning, Shaanxi, Qinghai, and Heilongjiang), and south (Shanghai, Yunnan, Sichuan, Anhui, Guangdong, Guangxi, Jiangsu, Jiangxi, Zhejiang, Hubei, Hunan, Fujian, Guizhou, and Chongqing) based on the Qinling Mountains-Huaihe River Line

**Table S4.** The association of TyG index with CMM after excluding individuals with DM.

| TyG index | Total  N | No. of Events  (Incident rate^#^) | **Model 1** | | **Model 2** | | **Model 3** | |
| --- | --- | --- | --- | --- | --- | --- | --- | --- |
|  |  |  | HR (95% CI) | *P* value | HR (95% CI) | *P* value | HR (95% CI) | *P* value |
| Continuous |  |  |  |  |  |  |  |  |
| Per 1.0 increase | 7606 | 518 (9.87) | 1.60 (1.42-1.79) | <0.001 | 1.28 (1.12-1.49) | <0.001 | 1.72 (1.40-2.11) | <0.001 |
| Quartiles |  |  |  |  |  |  |  |  |
| Q1 | 1902 | 73 (5.54) | Ref. |  | Ref. |  | Ref. |  |
| Q2 | 1901 | 103 (7.84) | 1.40 (1.04-1.88) | 0.026 | 1.35 (0.98-1.85) | 0.067 | 1.27 (0.92-1.76) | 0.150 |
| Q3 | 1902 | 154 (11.72) | 2.12 (1.61-2.80) | <0.001 | 1.60 (1.18-2.17) | 0.003 | 1.50 (1.10-2.06) | 0.011 |
| Q4 | 1901 | 188 (14.40) | 2.69 (2.05-3.52) | <0.001 | 1.72 (1.27-2.34) | 0.001 | 1.79 (1.28-2.51) | 0.001 |

Model 1: unadjusted

Model 2: adjusted for age, sex, SBP, DBP, BMI, alcohol consumption and smoking status

Model 3: model 2 + further adjusted for marital status, education, rural residence, heart rate, BUN, serum creatinine, UA, hemoglobin, TC, LDL, stroke, and heart disease

BMI, body mass index; BUN, blood urea nitrogen; CI, confidence interval; CMM, cardiometabolic multimorbidity; DBP, diastolic blood pressure; DM, diabetes mellitus; HR, hazard ratio; LDL, low density lipoprotein; Q, quartile; Ref, reference; SBP, systolic blood pressure; TC, total cholesterol; UA, uric acid

# Incident rate was presented as per 1000 person-years of follow-up

**Table S5.** The association of TyG index with CMM after excluding individuals with stroke.

| TyG index | Total  N | No. of Events  (Incident rate^#^) | **Model 1** | | **Model 2** | | **Model 3** | |
| --- | --- | --- | --- | --- | --- | --- | --- | --- |
|  |  |  | HR (95% CI) | *P* value | HR (95% CI) | *P* value | HR (95% CI) | *P* value |
| Continuous |  |  |  |  |  |  |  |  |
| Per 1.0 increase | 7860 | 610 (11.27) | 1.79 (1.63-1.97) | <0.001 | 1.54 (1.37-1.73) | <0.001 | 1.55 (1.29-1.85) | <0.001 |
| Quartiles |  |  |  |  |  |  |  |  |
| Q1 | 1965 | 79 (5.81) | Ref. |  | Ref. |  | Ref. |  |
| Q2 | 1965 | 115 (8.48) | 1.45 (1.09-1.93) | 0.011 | 1.39 (1.02-1.89) | 0.037 | 1.32 (0.96-1.81) | 0.087 |
| Q3 | 1965 | 165 (12.19) | 2.09 (1.60-2.73) | <0.001 | 1.56 (1.16-2.11) | 0.003 | 1.41 (1.03-1.91) | 0.030 |
| Q4 | 1965 | 251 (18.71) | 3.24 (2.51-4.17) | <0.001 | 2.13 (1.60-2.83) | <0.001 | 1.61 (1.17-2.22) | 0.004 |

Model 1: unadjusted

Model 2: adjusted for age, sex, SBP, DBP, BMI, alcohol consumption and smoking status

Model 3: model 2 + further adjusted for marital status, education, rural residence, heart rate, BUN, serum creatinine, UA, hemoglobin, TC, LDL, heart disease, and DM

BMI, body mass index; BUN, blood urea nitrogen; CI, confidence interval; CMM, cardiometabolic multimorbidity; DBP, diastolic blood pressure; DM, diabetes mellitus; HR, hazard ratio; LDL, low density lipoprotein; Q, quartile; Ref, reference; SBP, systolic blood pressure; TC, total cholesterol; UA, uric acid

# Incident rate was presented as per 1000 person-years of follow-up

**Table S6.** The association of TyG index with CMM after excluding individuals with heart disease.

| TyG index | Total  N | No. of Events  (Incident rate^#^) | **Model 1** | | **Model 2** | | **Model 3** | |
| --- | --- | --- | --- | --- | --- | --- | --- | --- |
|  |  |  | HR (95% CI) | *P* value | HR (95% CI) | *P* value | HR (95% CI) | *P* value |
| Continuous |  |  |  |  |  |  |  |  |
| Per 1.0 increase | 7218 | 451 (9.05) | 1.90 (1.70-2.11) | <0.001 | 1.62 (1.42-1.84) | <0.001 | 1.49 (1.22-1.82) | <0.001 |
| Quartiles |  |  |  |  |  |  |  |  |
| Q1 | 1805 | 60 (4.79) | Ref. |  | Ref. |  | Ref. |  |
| Q2 | 1804 | 83 (6.65) | 1.23 (0.87-1.75) | 0.239 | 1.23 (0.87-1.75) | 0.239 | 1.15 (0.81-1.65) | 0.439 |
| Q3 | 1805 | 115 (9.21) | 1.35 (0.96-1.89) | 0.085 | 1.35 (0.96-1.89) | 0.085 | 1.16 (0.82-1.64) | 0.413 |
| Q4 | 1804 | 193 (15.60) | 2.05 (1.49-2.82) | <0.001 | 2.05 (1.49-2.82) | <0.001 | 1.42 (0.99-2.04) | 0.056 |

Model 1: unadjusted

Model 2: adjusted for age, sex, SBP, DBP, BMI, alcohol consumption and smoking status

Model 3: model 2 + further adjusted for marital status, education, rural residence, heart rate, BUN, serum creatinine, UA, hemoglobin, TC, LDL, stroke, and DM

BMI, body mass index; BUN, blood urea nitrogen; CI, confidence interval; CMM, cardiometabolic multimorbidity; DBP, diastolic blood pressure; DM, diabetes mellitus; HR, hazard ratio; LDL, low density lipoprotein; Q, quartile; Ref, reference; SBP, systolic blood pressure; TC, total cholesterol; UA, uric acid

# Incident rate was presented as per 1000 person-years of follow-up

**Table S7.** The association of TyG index with CMM after excluding individuals with kidney disease.

| TyG index | Total  N | No. of Events  (Incident rate^#^) | **Model 1** | | **Model 2** | | **Model 3** | |
| --- | --- | --- | --- | --- | --- | --- | --- | --- |
|  |  |  | HR (95% CI) | *P* value | HR (95% CI) | *P* value | HR (95% CI) | *P* value |
| Continuous |  |  |  |  |  |  |  |  |
| Per 1.0 increase | 7525 | 570 (10.99) | 1.80 (1.63-2.00) | <0.001 | 1.53 (1.36-1.73) | <0.001 | 1.62 (1.34-1.95) | <0.001 |
| Quartiles |  |  |  |  |  |  |  |  |
| Q1 | 1882 | 73 (5.60) | Ref. |  | Ref. |  | Ref. |  |
| Q2 | 1881 | 103 (7.92) | 1.42 (1.05-1.91) | 0.023 | 1.35 (0.97-1.86) | 0.073 | 1.26 (0.90-1.75) | 0.180 |
| Q3 | 1881 | 161 (12.42) | 2.16 (1.63-2.85) | <0.001 | 1.64 (1.21-2.23) | 0.002 | 1.47 (1.07-2.03) | 0.017 |
| Q4 | 1881 | 233 (18.13) | 3.27 (2.51-4.25) | <0.001 | 2.17 (1.61-2.92) | <0.001 | 1.70 (1.21-2.37) | 0.002 |

Model 1: unadjusted

Model 2: adjusted for age, sex, SBP, DBP, BMI, alcohol consumption and smoking status

Model 3: model 2 + further adjusted for marital status, education, rural residence, heart rate, BUN, serum creatinine, UA, hemoglobin, TC, LDL, stroke, heart disease, and DM

BMI, body mass index; BUN, blood urea nitrogen; CI, confidence interval; CMM, cardiometabolic multimorbidity; DBP, diastolic blood pressure; DM, diabetes mellitus; HR, hazard ratio; LDL, low density lipoprotein; Q, quartile; Ref, reference; SBP, systolic blood pressure; TC, total cholesterol; UA, uric acid

# Incident rate was presented as per 1000 person-years of follow-up

**Table S8.** The association of TyG index with CMM after imputing the baseline missing values.

| TyG index | Total  N | No. of Events  (Incident rate^#^) | **Model 1** | | **Model 2** | | **Model 3** | |
| --- | --- | --- | --- | --- | --- | --- | --- | --- |
|  |  |  | HR (95% CI) | *P* value | HR (95% CI) | *P* value | HR (95% CI) | *P* value |
| Continuous |  |  |  |  |  |  |  |  |
| Per 1.0 increase | 7970 | 638 (11.63) | 1.76 (1.60-1.94) | <0.001 | 1.52 (1.37-1.69) | <0.001 | 1.54 (1.31-1.80) | <0.001 |
| Quartiles |  |  |  |  |  |  |  |  |
| Q1 | 1992 | 84 (6.17) | Ref. |  | Ref. |  | Ref. |  |
| Q2 | 1993 | 122 (8.87) | 1.44 (1.09-1.90) | 0.010 | 1.25 (0.94-1.65) | 0.125 | 1.16 (0.88-1.54) | 0.290 |
| Q3 | 1993 | 175 (12.75) | 2.08 (1.60-2.70) | <0.001 | 1.51 (1.16-1.97) | 0.002 | 1.36 (1.04-1.78) | 0.026 |
| Q4 | 1992 | 257 (18.91) | 3.13 (2.44-4.00) | <0.001 | 2.12 (1.65-2.73) | <0.001 | 1.65 (1.26-2.18) | <0.001 |

Model 1: unadjusted

Model 2: adjusted for age, sex, SBP, DBP, BMI, alcohol consumption and smoking status

Model 3: model 2 + further adjusted for marital status, education, rural residence, heart rate, BUN, serum creatinine, UA, hemoglobin, TC, LDL, stroke, heart disease, and DM

BMI, body mass index; BUN, blood urea nitrogen; CI, confidence interval; CMM, cardiometabolic multimorbidity; DBP, diastolic blood pressure; DM, diabetes mellitus; HR, hazard ratio; LDL, low density lipoprotein; Q, quartile; Ref, reference; SBP, systolic blood pressure; TC, total cholesterol; UA, uric acid

# Incident rate was presented as per 1000 person-years of follow-up
